# Anticipatory guidance and care in pediatric and adult neurology for people with epilepsy who became pregnant

**DOI:** 10.1016/j.yebeh.2025.110292

**Published:** 2025-02-20

**Authors:** Amy K. Tao, Jasmin Rivero-Guerra, Katherine N. McFarlane, Wesley T. Kerr, Page B. Pennell, Judy C. Chang, Traci M. Kazmerski, Elizabeth I. Harrison, Laura Kirkpatrick

**Affiliations:** a UPMC Children’s Hospital of Pittsburgh, 4401 Penn Avenue, Pittsburgh, PA 15224, United States; b Department of Pediatrics, University of Pittsburgh, 4401 Penn Avenue, Pittsburgh, PA 15224, United States; c Department of Neurology, University of Pittsburgh, 811 Kaufmann Medical Building, 3461 Fifth Avenue, Pittsburgh, PA 15213, United States; d Department of Obstetrics, Gynecology, and Reproductive Sciences, University of Pittsburgh, 300 Halket Street, Pittsburgh, PA 15213, United States; e Department of Internal Medicine, University of Pittsburgh, 3550 Terrace Street, Pittsburgh, PA 15261, United States

**Keywords:** Anti-seizure medication, Counseling, Pregnancy, Teratogenicity, Folic acid

## Abstract

**Objective:**

To assess documentation of pregnancy-related counseling and care for people with epilepsy of childbearing potential (PWECP) in pediatric and adult neurology who became pregnant.

**Methods:**

We reviewed health records for primigravida PWECP prescribed an antiseizure medication (ASM) who delivered between June 2014 and May 2024 within one academic medical center. We used chi-squared tests to compare counseling, ASM prescriptions, and recommendations for supplemental folic acid between individuals in pediatric and adult neurology care before pregnancy. We performed logistic regression for these outcomes of pre-pregnancy counseling associated with type of neurology care, race, ethnicity, intellectual disability (ID), teratogenic profile of ASMs prescribed, and ASM polytherapy.

**Results:**

173 PWECP (84 % White non-Hispanic, 9 % with intellectual disability (ID) were included. Twenty-one (12 %) transferred from pediatric to adult neurology care due to pregnancy (“pediatric group”) and 152 (88 %) were previously established with adult neurology (“adult group”). PWECP in the pediatric group compared to the adult group had lower rates of documentation of clinician discussion of ASM teratogenicity (43 % vs 66 %, p = 0.041) and folic acid use (24 % vs 63 %, p = 0.001) before pregnancy. PWECP established with adult neurology prior to pregnancy were significantly more likely to have been taking folic acid before pregnancy (OR 5.21, 95 % CI 1.78–15.3). Individuals with ID were significantly less likely to have documentation of discussion of ASM teratogenicity (OR 0.18, 95 % CI 0.05–0.62).

**Conclusion:**

Our findings suggest a need for improvement in providing pre-pregnancy guidance and care for PWECP, especially for PWECP in pediatric neurology care and those with ID.

## 1. Introduction

People with epilepsy of childbearing potential (PWECP) taking antiseizure medication (ASM) are at risk of adverse outcomes in their offspring [1], including congenital malformations and adverse neuro-developmental effects [2-5]. Daily folic acid supplementation is associated with improved neurodevelopmental outcomes in children born to PWECP [4-9]. In practice guidelines published in 2024, the American Academy of Neurology recommends that clinicians consider using low risk ASMs when appropriate to minimize the risk of major fetal congenital malformations, prescribe daily folic acid supplementation, and counsel PWECP taking ASMs that adherence to recommended folic acid supplementation is important to promote optimal neuro-developmental outcomes [10]. Anticipatory guidance in advance of pregnancy is important for all PWECP regardless of whether they plan or desire to become pregnant, as over half of pregnancies among PWECP are reported to be unintended [11,12].

Many studies identify gaps in reproductive healthcare and counseling for PWECP in both pediatric and adult neurology [13-17]. However, limited literature has evaluated counseling prior to pregnancy in individuals who later became pregnant. Moreover, few studies have compared rates of guidance and care prior to first pregnancy between pediatric and adult neurology, an area important for study as rates of unintended pregnancy are highest for people with epilepsy less than 18 years of age, similar to the general population [12,18]. In this retrospective study of electronic health records, we compared documentation of pre-pregnancy counseling and care between PWECP who transferred from pediatric to adult neurology care due to becoming pregnant (as is standard practice in our institution) and those who had established care with adult neurology prior to pregnancy. We hypothesized that PWECP seen by pediatric neurology prior to pregnancy received appropriate pre-pregnancy guidance and care at lower rates than PWECP established with adult neurology prior to pregnancy.

## 2. Methods

### 2.1. Study Design & data

In collaboration with a clinical informatics service, we obtained a list of all primigravida individuals who had an encounter in one academic medical center, who were given an ICD diagnosis code consistent with epilepsy (G40.*) and who delivered between June 1, 2014 and May 31, 2024. We used this method of patient identification given that it has a sensitivity of 88 %, specificity of 98 %, and area under the curve of 93 % in other similar datasets [19]. We excluded PWECP who were not prescribed an ASM at time of pregnancy diagnosis or had no clinician documentation of topics discussed at outpatient clinic visits.

### 2.2. Chart review

We manually reviewed electronic health record documentation from neurology outpatient clinic and visits with a prenatal clinician. We confirmed the diagnosis of epilepsy and excluded PWECP deemed to not have epilepsy in subsequent chart review (for example, if medical record documentation indicated that they had functional but not epileptic seizures). We collected demographic information based on chart documentation, including whether the individual had intellectual disability (ID) and drug-resistant epilepsy based on International League Against Epilepsy criteria [20].

Within our institution, transition of care from pediatric to adult neurology is at the discretion of the patient and clinician for patients between 18 and 26 years of age, with the exception of patients who become pregnant who must be transferred to adult care. We determined whether PWECP in our study were established with adult neurology prior to pregnancy or were only seen by a pediatric neurology clinician prior to pregnancy and transferred to adult neurology due to becoming pregnant.

We extracted information from clinician notes prior to pregnancy for the following outcomes of receiving pregnancy-related anticipatory guidance:

1. Teratogenic risk of ASM(s) prescribed at time of pregnancy diagnosis, defined by association with an increased risk of major congenital malformation events from the International Registry of Antiepileptic Drugs and Pregnancy study [2]
  - Teratogenic ASMs: carbamazepine, phenobarbital, phenytoin, topiramate, valproate
  - Lower risk ASMs: lamotrigine, levetiracetam, oxcarbazepine
  - ASMs with unknown risk included all other ASMs
2. Clinician documentation of discussion prior to pregnancy about teratogenic risks of ASMs or relative safety of ASMs for fetal development
3. Clinician documentation of recommendation for daily supplemental folic acid and/or prescription for supplemental folic acid prior to pregnancy
4. Clinician documentation of PWECP taking supplemental folic acid prior to pregnancy in records from outpatient visits before pregnancy or initial prenatal visit
5. PWECP adherence to folic acid recommendation prior to pregnancy, based on documentation of PWECP taking supplemental folic acid before pregnancy or initial prenatal visit if clinician documented recommendation and/or prescription for folic acid

### 2.3. Statistical Analysis

We calculated descriptive statistics and compared categorical outcomes of documentation of pre-pregnancy care and guidance between PWECP who transferred from pediatric to adult neurology due to becoming pregnant and those who had established care with adult neurology prior to pregnancy using a chi-squared test or Fisher Exact test as appropriate. We then performed logistic regression for these outcomes of pre-pregnancy care and guidance to account for pediatric vs adult neurology care, race and ethnicity (white non-Hispanic vs other race or ethnicity), ID, ASM teratogenicity, and ASM polytherapy at time of pregnancy diagnosis. We did not include age as a variable due to its collinearity with transfer of care. We conducted statistical analyses in STATA version 17.0 (StataCorp, College Station, Texas).

### 2.4. IRB Approval

The University of Pittsburgh Institutional Review Board deemed this study exempt from review.

## 3. Results

### 3.1. Demographics

Of the 173 PWECP meeting inclusion criteria, 21 (12 %) transferred from pediatric to adult neurology due to pregnancy (“pediatric group,” median age at delivery 20 years, interquartile range (IQR) 18–21, range 13–25) and 152 (88 %) had established care with adult neurology prior to pregnancy (“adult group,” median age at delivery 28 years, IQR 24–31, range 19–41). Most PWECP identified as White (87 %) and non-Hispanic/Latino(a) (84 %). ID was documented for 16 individuals (9 %). Four (19 %) and 30 (20 %) individuals in the pediatric and adult groups respectively had drug-resistant epilepsy.

Detailed demographics are listed in Table 1.

### 3.2. ASM teratogenicity and discussion of teratogenicity

Nineteen percent of the pediatric group and 14 % of the adult group were prescribed an ASM with known teratogenic effects prior to pregnancy diagnosis (p = 0.52). Fifty-two percent of the pediatric group and 66 % of the adult group were prescribed only lower risk ASMs prior to pregnancy diagnosis (p = 0.21). ASMs with unknown risks prescribed for PWECP included in the study were brivaracetam, clonazepam, eslicarbazepine, ethosuximide, gabapentin, lacosamide, perampanel, pregabalin, and zonisamide. Topiramate was prescribed for 4 and 10 PWECP in the pediatric (19 %) and adult (7 %) groups respectively. No other ASMs with known teratogenic potential were prescribed for PWECP in the pediatric group. Other teratogenic ASMs prescribed for PWECP in the adult group included carbamazepine for 6 individuals (4 %), valproate for 4 individuals (3 %), phenobarbital for 1 individual (0.7 %), and phenytoin for 1 individual (0.7 %). Documented discussion of teratogenic risks of ASMs prior to pregnancy was lower in the pediatric group (43 %) than the adult group (66 %, p = 0.041).

### 3.3. Folic acid supplementation

Clinicians documented recommendation for supplemental folic acid for 62 % of the pediatric group and 72 % of the adult group (p = 0.32). There were lower rates of documentation of PWECP taking supplemental folic acid prior to pregnancy in the pediatric group than the adult group (24 % vs 63 %, p = 0.001). Of PWECP recommended folic acid supplemental prior to pregnancy (pediatric group n = 13, adult group n = 110), PWECP in the pediatric group had documentation of adherence to the recommendation at lower rates (38 % vs 87 %, p < 0.001) than those in the adult group.

### 3.4. Logistic regression

PWECP who were established with adult neurology prior to pregnancy were significantly more likely to have documentation of taking folic acid prior to pregnancy (OR 5.19, 95 % CI 1.77–15.19, p = 0.003), significantly more likely to have adhered to taking supplemental folic acid if recommended (OR 11.63, 95 % CI 3.04–44.33, p < 0.0001), and significantly more likely to have documentation of discussion of ASM teratogenicity (OR 2.78, 95 % CI 1.03–7.44, p = 0.042) (Table 2). PWECP with ID were significantly less likely to have been prescribed only lower-risk ASMs (OR 0.25, 95 % CI 0.06–0.88, p = 0.031), significantly less likely to have adhered to the folic acid recommendation if recommended (OR 0.14, 95 % CI 0.02–0.73, p = 0.019), and significantly less likely to have received discussion of ASM teratogenicity (OR 0.18, 95 % CI 0.05–0.65, p = 0.008) than those without ID (Table 2). PWECP on ASM polytherapy prior to pregnancy diagnosis were significantly more likely to be taking a teratogenic ASM (OR 4.72, 95 % CI 1.73–12.85, p = 0.002) and significantly less likely to be prescribed only lower-risk ASMs (OR 0.12, 95 % CI 0.05–0.27, p < 0.0001) (Table 2). PWECP taking a teratogenic ASM prior to pregnancy diagnosis were less likely to have received clinician recommendation for supplemental folic acid prior to pregnancy (OR 0.31, 95 % CI 0.11–0.79, p = 0.014). PWECP with drug-resistant epilepsy were significantly less likely to have been prescribed only lower-risk ASMs (OR 0.35, 95 % CI 0.13–0.93, p = 0.035) (Table 2).

## 4. Discussion

Our study findings identify a need for improvement in both pediatric and adult neurology in providing pregnancy-related care and counseling prior to pregnancy for PWECP prescribed an ASM. PWECP in our study had suboptimal rates of lower risk ASM prescription, folic acid recommendation, adherence to the folic acid recommendation, and discussion about ASM teratogenicity prior to pregnancy. Improvement is particularly needed for individuals in pediatric neurology care and those with ID.

Past qualitative studies identified that PWECP value and desire counseling about the impact of epilepsy on sexual and reproductive health beginning in adolescence, even if they are not actively planning for or pursuing pregnancy [21]. PWECP want clinicians to initiate conversations about reproductive health beginning in adolescence, but report receiving ineffective counseling, especially related to folic acid supplementation and teratogenic medications [22]. PWECP have limited knowledge about the teratogenic effects of ASMs [23]. Therefore, neurologists must provide proper counseling and guidance, emphasizing the reproductive risks associated with epilepsy and ASMs prescribed if they become pregnant and actions that they can take to promote healthy pregnancy.

Our findings indicate a need for improvement in both pediatric and adult neurology in preparing all PWECP for the possibility of pregnancy and providing anticipatory guidance and care. Since 1998 (years before individuals in our study became pregnant), American Academy of Neurology practice guidelines recommend that neurologists discuss pre-pregnancy counseling with PWECP, including topics of folic acid supplementation and teratogenic potential of ASMs [10,24]. Despite these guidelines, our findings identify areas for improvement in providing this counseling, particularly among pediatric neurologists. Multiple individuals in our study had been prescribed an ASM with known teratogenic risks, but did not have documentation of discussion about ASM teratogenicity and, therefore, may have been unaware of the teratogenic potential of their medication(s). Improvement in these aspects of care is of critical importance for the pediatric population of PWECP considering rates of unintended pregnancies in PWECP are highest in younger age groups [17].

Various barriers may prevent clinicians from providing pregnancy-related counseling, including limited time and other competing topics of discussion during clinic visits, patient or clinician discomfort, and limited incorporation of sexual and reproductive health content in pediatric neurology training, as identified in a qualitative study of pediatric neurologists and epileptologists [25]. Our results also indicate a need to explore barriers to PWECP adherence to supplemental folic acid recommendations, especially in pediatric neurology.

Neurologists also must be cognizant of providing proper guidance and counseling to PWECP with ID, which was documented in 9 % of individuals in the study. Our findings support results of past studies that identified variable and, at times, suboptimal practices and knowledge in clinicians regarding reproductive healthcare for PWECP with ID [13,26]. The rate of ID in PWECP in this study (9 %) is lower than previous estimates, as approximately 17 % of people with epilepsy had ID in a recent epidemiological study [27] and 25 % within people with epilepsy seen in our institution have ID. This finding suggests that PWECP with ID become pregnant less often than those without ID. Nonetheless, improvement in providing proper pre-pregnancy guidance to this population is important given high rates of co-occurrence between epilepsy and ID [28].

Limitations of this study include that we examined clinician documentation of folic acid supplementation and ASM teratogenicity as an indicator of whether pre-pregnancy care and guidance was provided, though clinicians might counsel PWECP on these topics without documenting the discussion. However, the documentation reviewed is a reasonable representation of anticipatory guidance provided, as no PWECP were reported to be taking supplemental folic acid without documentation of the clinician recommending supplementation. Clinician documentation was only utilized to assess whether certain measures of guidance and care were provided and does not capture the extent or quality of counseling. In addition, this study occurred at a single academic medical center which may limit generalizability, although suboptimal awareness regarding reproductive healthcare for PWECP has been reported among national samples of healthcare clinicians [13,25]. Racial and ethnic diversity is also limited among the PWECP studied, which reflects the population of western Pennsylvania where the study was conducted and may impact the generalizability of these findings.

Areas rich for future study include assessing the quality of pregnancy-related counseling instead of merely documentation of its occurrence, planning interventions to increase rates of clinician counseling and PWECP adherence, and comparing pediatric and adult neurology care of PWECP at multiple institutions. Actionable measures for improvement include targeted interventions to increase clinician awareness and adherence to AAN guidelines for care of PWECP which may include professional training, patient education, and health systems changes. Our study findings highlight specific needs for improvement in neurology care of PWECP in advance of pregnancy, including prescription of lower-risk ASMs when appropriate, counseling on the teratogenic risks of ASMs, and recommendation for folic acid supplementation.

## Figures and Tables

**Table 1:** Demographics.

Demographic variable	n (%) or median (IQR, range)		
	Pediatric group (n = 21)	Adult group (n = 152)	Total (n = 173)
Race			
White	17 (81 %)	133 (88 %)	150 (87 %)
Black/African American	2 (10 %)	15 (10 %)	17 (10 %)
Asian	0 (0 %)	4 (2 %)	4 (2 %)
American Indian/Alaskan Native	1 (5 %)	0 (0 %)	1 (0.5 %)
Unknown	1 (5 %)	0 (0 %)	1 (0.5 %)
Ethnicity			
Non-Hispanic or Latino/a	19 (90 %)	149 (98 %)	168 (97 %)
Unknown/unreported	2 (10 %)	3 (2 %)	5 (3 %)
Age of PWECP at delivery	20 (IQR 18–21, range 13–25)	28 (IQR 24–31, range 19–41)	27 (IQR 23–31, range 13–41)
Type of epilepsy			
Generalized	11 (52 %)	77 (51 %)	88 (51 %)
Focal	5 (24 %)	59 (39 %)	64 (37 %)
Unknown	5 (24 %)	16 (11 %)	21 (12 %)
Drug-resistant epilepsy	4 (19 %)	30 (20 %)	34 (20 %)
Intellectual disability	2 (10 %)	13 (9 %)	15 (9 %)
Risk of Prescribed ASMs at Pregnancy Diagnosis			
Teratogenic ASM(s)	4 (19 %)	21 (14 %)	25 (14 %)
ASM(s) with unknown risk	6 (29 %)	30 (20 %)	36 (21 %)
Only low risk ASM(s)	11 (52 %)	101 (66 %)	112 (65 %)
Change in ASMs prescribed between pregnancy diagnosis and delivery	6 (29 %)	31 (20 %)	37 (21 %)

Demographics for individuals who transferred from pediatric to adult neurology care due to pregnancy (“pediatric group”), individuals who were previously established with adult neurology (“adult group”), and all PWECP in the study.

**Table 2:** Logistic Regression for Key Outcomes.

	Established with Adult Neurology		Non-white Hispanic race and ethnicity		Intellectual Disability		Teratogenic ASM at Diagnosis		Polytherapy at Diagnosis		Drug-resistant epilepsy	
						
	OR	95 % CI	OR	95 % CI	OR	95 % CI	OR	95 % CI	OR	95 % CI	OR	95 % CI
Recommended Folic Acid Prior to Pregnancy	1.44	(0.54–3.83)	0.71	(0.28–1.75)	1.05	(0.31–3.55)	*0.31	(0.11–0.79)	1.80	(0.73–4.41)	1.34	(0.48–3.70)
Took Folic Acid Prior to Pregnancy	*5.19	(1.77–15.19)	0.60	(0.24–1.46)	0.45	(0.13–1.43)	0.60	(0.23–1.54)	1.79	(0.78–4.08)	0.86	(0.34–2.12)
Adhered to Folic Acid Recommendation Prior to Pregnancy if recommended	*11.6	(3.04–44.33)	0.41	(0.10–1.64)	*0.14	(0.02–0.73)	5.25	(0.43–63.00)	1.69	(0.37–7.68)	0.57	(0.12–2.62)
Teratogenic ASM Prior to Pregnancy Diagnosis	0.57	(0.16–1.98)	1.14	(0.31–4.07)	3.03	(0.87–10.52)	N/A	N/A	*4.72	(1.73–12.85)	0.49	(0.14–1.65)
Only Lower Risk ASM(s) Prior to Pregnancy Diagnosis	2.90	(0.97–8.57)	1.75	(0.53–5.73)	*0.25	(0.06–0.88)	N/A	N/A	*0.12	(0.05–0.27)	*0.35	(0.13–0.93)
Documented Discussion of Teratogenicity Prior to Pregnancy	*2.78	(1.03–7.44)	0.43	(0.17–1.05)	*0.18	(0.05–0.65)	1.30	(0.46–3.59)	0.70	(0.30–1.63)	2.59	(0.93–7.20)

* Denotes statistical significance at an alpha level of p = 0.05.

## Abbreviations:

PWECP: people with epilepsy of childbearing potential
ASM: antiseizure medication
ID: intellectual disability
